# The impact of climate change on the lives and livelihoods of readymade garment (RMG) workers: an exploratory study in selected readymade garment factories in Bangladesh

**DOI:** 10.1186/s12889-023-17165-7

**Published:** 2023-11-20

**Authors:** Mohammad Hayatun Nabi, Mehedi Hasan, Anika Tasneem Chowdhury, Farah Naz, Mosharop Hossian

**Affiliations:** 1 Department of Public Health, North South University, Dhaka, Bangladesh; 2 Maternal and Child Health Division, International Centre for Diarrhoeal Disease Research, Dhaka, Bangladesh; 3 Department of Physiology, Green Life Medical College, Dhaka, Bangladesh; 4 Public Health Promotion and Development Society (PPDS), Dhaka, Bangladesh; 5 School of Health and Rehabilitation Sciences, The University of Queensland, St Lucia, Australia

**Keywords:** Climate Change, Ready made Garment Industry, Bangladesh, Climate Change Impact

## Abstract

**Background:**

There is a paucity of resources focusing on the climate change experience of readymade garment (RMG) workers in developing countries such as Bangladesh. Therefore, this mixed method approach aims to understand the distinctive types of climate change experiences from a health and occupational perspective, along with the consequences of these changes among RMG workers in Bangladesh.

**Methods:**

The study was conducted from January 2022 and February 2022 where the quantitative data were collected from 200 RMG workers in 10 randomly selected garments and two focus group discussions took place with 20 conveniently selected RMG workers. The key informants were relevant stakeholders in the industry. Quantitative findings were reported using descriptive methods and qualitative findings were analysed using a content analysis approach.

**Result:**

A total of 200 RMG workers were included in the study of which the majority belonged to the age group of 26–30 years (44%), were male (55%), worked in a compliant factory (70%), and were machine operators (79%). Half of the respondents experienced damage from natural disasters (51%), but only approximately 37% received humanitarian help. Migration and urbanisation were among the aftermath of the damage caused by e natural disasters, and 42% were forced to shift their homes due to natural disasters. Competition in the job market increased, and the owners had the opportunity to take on employees at a reduced salary. The respondents flagged climate change as a major contributor to their disease patterns. More than three-quarters of the respondents became sick because of increased heat while working; however, only half received any treatment.

**Conclusion:**

Employee participation in hazard recognition, employer preparedness, prevention through design, research, surveillance, and upholding workplace ethics and standards can be the answers to climate change problems for readymade garment workers.

## Introduction

In 2020, a report stated that 2010 to 2019 was the hottest decade in the last 140 years and 2020 was the warmest year since record-keeping began drawing the attention of the world to the undeniable realities of climate change and it is happening now [1, 2]. Climate change and its negative impacts encompass the globe, but are concentrated in poor countries, although they contribute the least [3, 4]. Consequently, developing countries such as Bangladesh suffer the worst from the impacts of climate change, as highlighted by several leading figures working against climate change [5]. According to the World Bank, more than 140 million people from sub-Saharan Africa, South Asia, and Latin America are predicted to be affected by climate change by 2050 [6]. In 2019 alone, 24.9 million weather-related displacements occurred in locations with frequent weather-related catastrophes, such as Bangladesh [3]. Such drastic demographic shifts can not only potentiate fragile national security and development but also reverse decades of achievements and, in turn, spread international unrest [7].

In 2017, Germanwatch Global Climate Risk Index reported that Bangladesh is in the top 10 most affected countries by climate change and as mentioned in the Johannesburg Declaration on Sustainable Development, Bangladesh is prone to a major setback in its ascent to economic stability [3, 8]. The economic burden is greatest for populations living near or below the poverty line, and the aftermath of any climate change can appear in the form of migration, loss of means of livelihood, and lack of job opportunities owing to increased economic vulnerability and damage to physical properties [9]. Adverse health outcomes, ranging from accidental incapacitation to psychological stress, cardiovascular and respiratory ailments, and chronic diseases including cancer and kidney diseases, can be traced back to the consequences of climate change in Bangladesh [10–12]. Owing to the severe lack of timely social and economic support, food shortages, safe drinking water, habitat, sanitation, and local civic amenities exacerbate the suffering of the poor and vulnerable populations [8, 13, 14].

Accordingly, the condition of Readymade Garments (RMG) workers in Bangladesh is particularly distressing, as 90% of the approximately five million are women who rely heavily on their income, positioning them among the worst affected by the predicted fall in GDP due to climate change, according to the Asian Development Bank [15, 16]. This sector contributes more than 23% of the country’s total GDP and 79.60% of the industrial sector’s contribution to the national GDP [17, 18]. Despite all the achievements, below average wages, job dissatisfaction and insecurity, frequent work accidents resulting from low safety measures, narrow staircases, overall substandard factory infrastructure, unhealthy work environment with inadequate light and ventilation, heat regulatory mechanisms, overcrowding, and many other factors continue to burden RMG workers [19–21].

Despite the fashion industry undergoing severe criticism and scrutiny for its failure to address the impacts of climate change worldwide, the amount of research on the impact of climate change on RMG workers in Bangladesh remains remarkably limited [22–24]. Readymade Garment (RMG) workers play a crucial role in sustaining this nation’s economy. However, their experiences regarding the consequences of climate change have been underinvestigated. Existing literature covers a range of topics related to worker well-being and resilience, including occupational safety, industrial disasters, housing conditions, heat management strategies, and the sustainability of human resources and supply chains. However, research on the impact of climate change on RMG workers in Bangladesh is limited [24–28]. Currently, policymakers and key stakeholders are at a binding to take proper measures to address climate change challenges owing to the dearth of evidence in this regard. This study was undertaken to address the lack of recent and overall research on unique climate change experiences and their health and occupational implications among RMG workers in Bangladesh. We anticipate that the findings of this study will demonstrate the adverse effects of climate change on the lives and livelihoods of RMG workers to policymakers related to both the RMG industry and the health sector so that they can work synergistically to formulate an appropriate plan and create the best working conditions for workers in this industry.

## Methodology

### Study design and study settings

As a symbol of best practice, quantitative and qualitative paradigms were brought together to generate research evidence; this process is also known as pragmatic mixed-method research design. In this case, we implemented a mixed-method study design based on recommendations from other researchers as it could stimulate in-depth knowledge of the underlying issue [29, 30]. Dhaka is the capital city of Bangladesh, and because the major garment factories in Bangladesh are located here, we selected 10 garment factories at the business center of Dhaka as our study site.

### Sampling technique and sample size

Our study included both male and female workers in the readymade garment industry. The participants were selected using a convenience sampling technique, ensuring they met the following criteria: they were over 18 years old, had at least one year of work experience in a garment factory, and were willing to provide consent to participate in the study. This study was conducted in several stages, as follows. Initially, we selected ten garment factories across Dhaka City using a probabilistic sampling method. Our calculated sample size was 191, with 88% prevalence of experiencing climate change among manual labour workers in Bangladesh, 5% error margin, and 15% attrition rate [31]. Survey data were collected from a total of 200 participants. Within each factory, we employed convenience sampling to select individual participants. We ensured an equal distribution of participants across the 10 factories. The sample size distribution is presented in Table 1.

**Table 1:** Sample size for quantitative data collection

S/N	Name of garments factory	Percentage to total sample size	Sample for each factory
1	Garment Factory 1	10%	20
2	Garment Factory 2	10%	20
3	Garment Factory 3	10%	20
4	Garment Factory 4	10%	20
5	Garment Factory 5	10%	20
6	Garment Factory 6	10%	20
7	Garment Factory 7	10%	20
8	Garment Factory 8	10%	20
9	Garment Factory 9	10%	20
10	Garment Factory 10	10%	20
	**Total**	100%	**200**

We conducted two separate focus group discussion (FGD) sessions: one with male participants and the other with female participants. Participants were invited from one of the initially selected factories through a lottery system. Those who agreed to participate in the interviews were enrolled in this study. For key informant interviews (KIIs), trade union activists, Bangladesh Garment Manufacturers and Exporters Association (BGMEA) representatives, public health experts, environmentalists, and representatives of relevant ministries were interviewed. Key informants were selected based on their work experience as experts at the policy level for more than five years and their accessibility and availability for the interviews. The sample size distributions of FGDs and KIIs are listed in Table 2.

**Table 2:** Sample size of FGD and KII

Method	Respondent	Number of participants
Focus group discussion	Readymade garment workers	20
Key informant interview	Trade union activist of the RMG sector	1
	Representative of Bangladesh Garment Manufacturer and Exporters Association (BGMEA)	1
	Environmentalist	1
	Representative of Ministry of Environment, Forest and Climate Change, Bangladesh	1
	Representative of Ministry of Labour, Bangladesh	1
	Public Health Expert	1
	**Total**	**6**

### Data collection

The data collection tools were interviewer-administrated questionnaires for the surveys and semi-structured guidelines for qualitative interviews. A quantitative questionnaire was designed specifically for readymade garment workers with the aim of gathering their experiences regarding climate change and included items relating to socioeconomic and demographic factors, damage patterns, societal response, impact of job, and emerging health issues due to climate change. A group of five experts, including a public health expert, medical doctor, climate change researcher, sociologist, and statistician thoroughly reviewed the questionnaire. Drawing on their unique experiences and expertise, they recommended paraphrasing and rearranging questions to ensure a smooth flow and easier understanding of the respondents. Before the main study, we conducted a pilot test with a group of 20 participants similar to those in the main study. This helped us verify the effectiveness of our questionnaire and make the necessary adjustments. We excluded the pilot participants from the main study to ensure reliability. In addition to the questionnaire, semi-structured interview guidelines were prepared for the Focus Group Discussions (FGDs) and Key Informant Interviews (KIIs). These guidelines served to delve deeper into the perceived effects of climate change on the lives and livelihoods of RMG workers. A group of qualified data collectors trained in both quantitative and qualitative data collection was assigned for the final data collection, which was conducted between January and February 2022.

### Data analysis

For our study, we adopted a thorough and methodical approach to data cleaning, structuring, and analysis using Statistical Package for Social Sciences (SPSS) software version 27. Since this was an exploratory study, we presented our study findings using descriptive statistics using mean and standard deviation for continuous variables, and frequency and percentage for qualitative variables. In terms of our qualitative data, we started by transcribing the primary patterns we observed during the Focus Group Discussions (FGDs) and Key Informant Interviews (KIIs). We then employed conventional content analysis to structure and analyse the data. This involved reading transcriptions multiple times, identifying key concepts, and categorising them into themes. Two distinct coding frames were developed to ensure a thorough understanding of the data. One coding frame was designed for the KII transcripts and the other for the FGD transcripts. These coding frames helped us prepare and finalise the themes, sub-themes, and our comprehensive codebook for content analysis. Once we completed the data analysis phase for both the quantitative and qualitative parts of our study, we integrated the findings using a convergent parallel database variant mixed-methods design. This design allowed us to triangulate the findings of our quantitative and qualitative data, helping us to understand where these different data collection methods diverge or converge. This comprehensive approach allows us to derive meaningful inferences from our findings.

## Results

Although, the qualitative data collection preceded the quantitative data collection, we triangulated both type of data to explain the study findings during the analysis period. Therefore, we first presented the quantitative data and later included supporting qualitative findings and vice versa as appropriate to narrate the result.

### Background characteristics and vocational status of the respondents

**Fig. 1:**
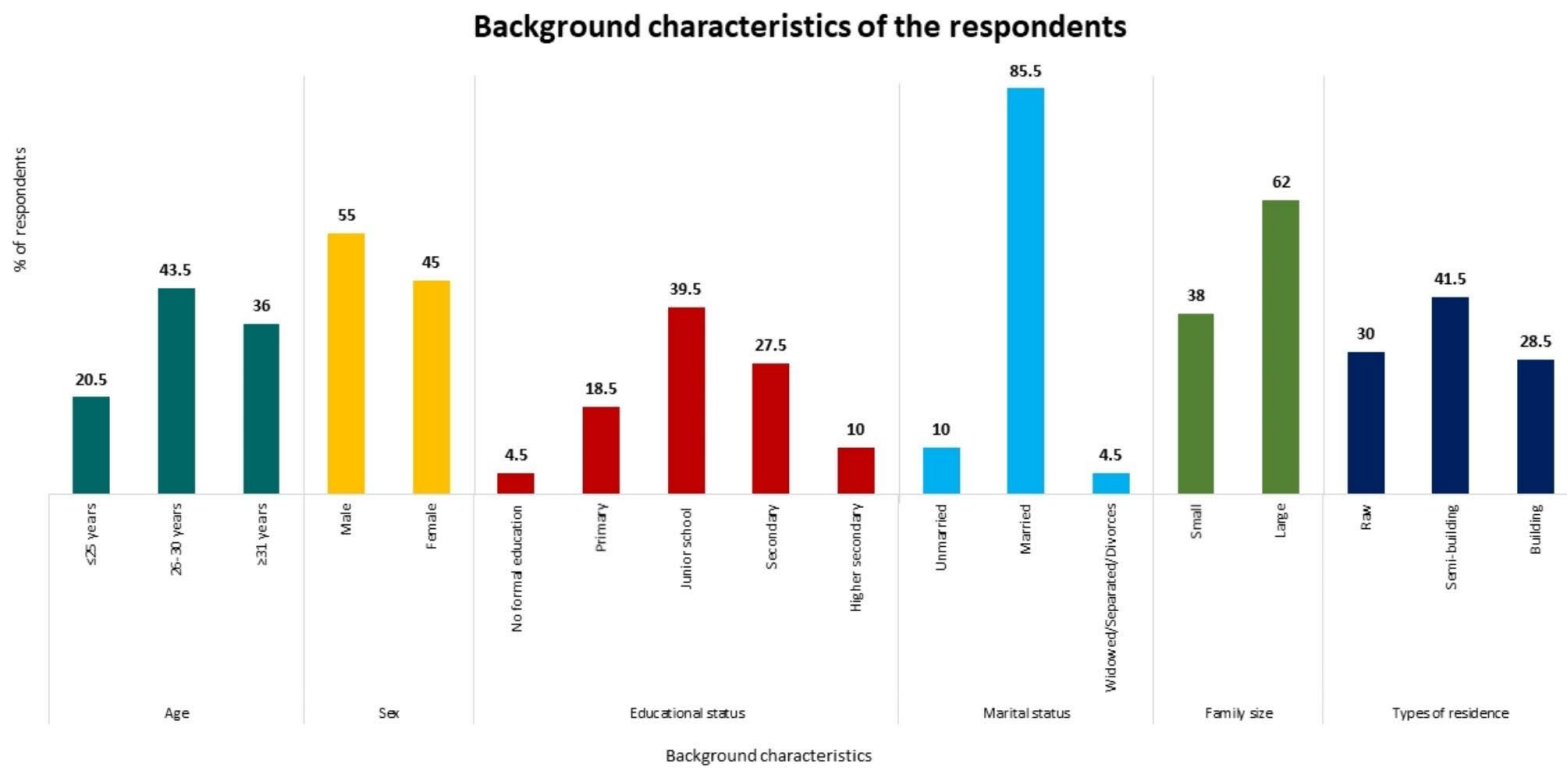
Background characteristics of the study respondents

In total, 200 RMG workers were included in this study. Figure 1 shows the the participants’ background characteristics. The majority of the respondents belonged to the 26–30 year age group (43.5%), were male (55%), and had a junior school certificate (39.5%). More than three-quarters of the participants were married (85.5%), more than half lived with a large family (62%), and more than one-quarter lived in a semi-building (41.5%).

Table 3 shows the vocational status of the study respondents. 70% of the respondents worked in a compliant factory, 79% worked as a machine operator, around 58% earned 10,001–15,000 BDT (93.40–140.08 USD) per month, and 45% had a work experience of 6–10 years [32].

**Table 3:** Vocational status of the respondents

Vocational status	Frequency (n)	Proportion (%)
**Factory type**		
Compliant	140	70
Non-compliant	60	30
**Designation**		
Helper	3	1.5
Trainee operator	18	9
Operator	158	79
Cutting/Finishing professional	21	10.5
**Monthly income (BDT)**		
≤ 10,000	21	10.5
10,001–15,000	115	57.5
≥ 15,001	64	32
**Duration of professional life**		
≤ 5 years	64	32
6–10 years	90	45
≥ 11 years	46	23

The impact of climate change is presented in two major themes: damage and risk management and impact on health. The minor themes under damage and risk management were migration as an aftermath of damage due to natural disasters and impact on job sector as an aftermath of the burden of migration.

### Damage and risk management

Table 4 describes the damage and risk management experienced by respondents due to climate change. Half of the respondents experienced damage from natural disasters (50.5%), the majority of whom faced flooding (79.9%), followed by drought (52.2%), river erosion (49.5%), earthquakes (45.1%), storm surges (39.7%), and landslides (13%). However, less than half received humanitarian assistance (36.6%).

**Table 4:** Damage and risk management experienced by the respondents due to climate change

Variables	Frequency (n)	Proportion (%)
**Damage experienced due to natural disasters in the last 10 years**		
Yes	101	50.5
No	99	49.5
**Types of natural disasters experienced (Multiple responses taken)**		
Flood	147	79.9
Drought	96	52.2
River erosion	91	49.5
Earthquake	83	45.1
Storm Surge	73	39.7
Landslide	24	13
**Received humanitarian help**		
Yes	37	36.6
No	64	63.4

### Factors influencing migration

The qualitative interviews revealed that migration and urbanisation were among the aftermath of the damages caused by natural disasters.

My uncle had to come to the city as river erosion took his home. He has six children. Three of them could find a job, but three are unemployed still.

(Male RMG Worker)

**Fig. 2:**
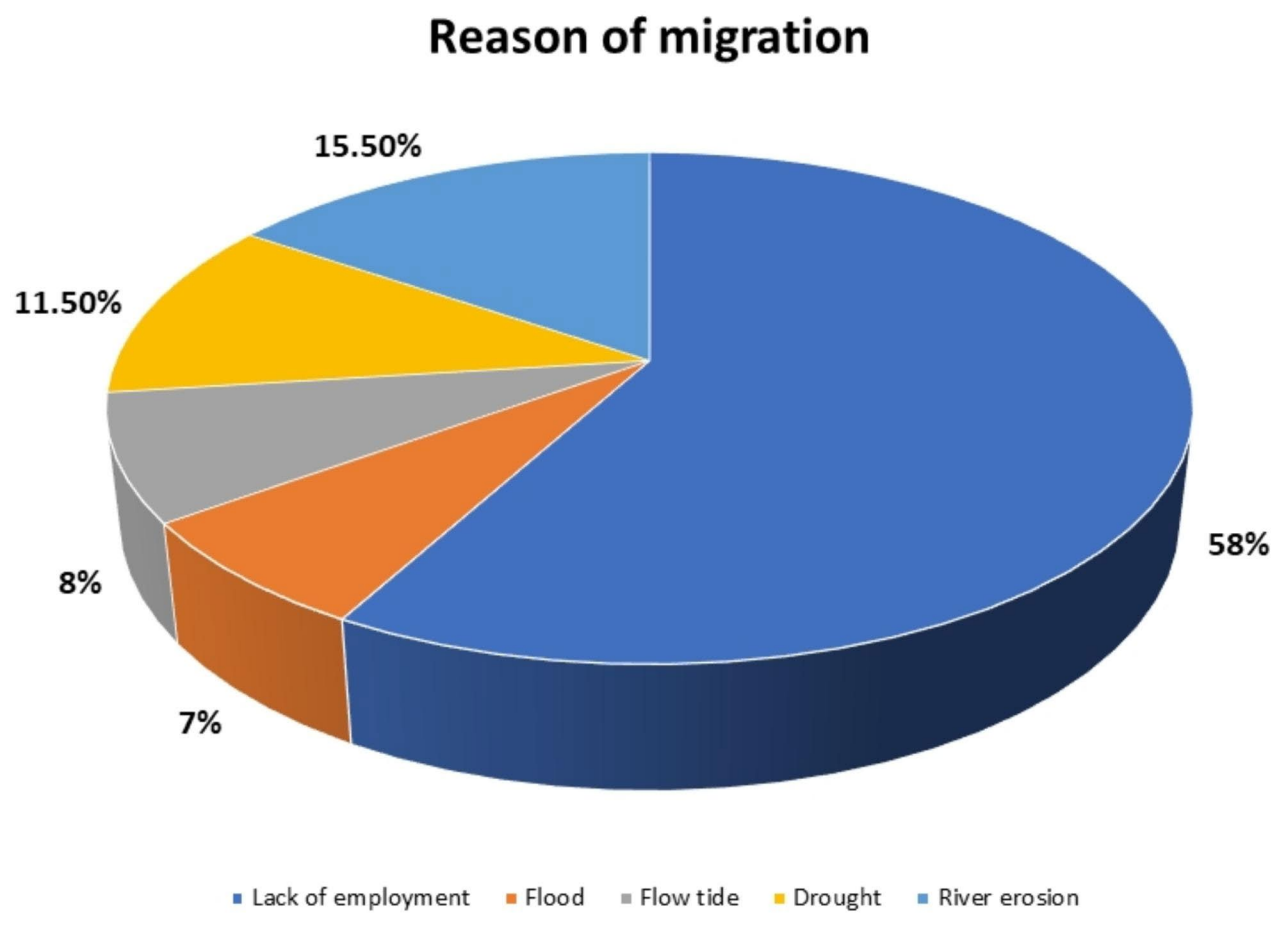
Reason for migration of the RMG workers

Figure 2 shows the reasons for the migration of RMG workers. While 58% of the respondents mentioned that job unavailability was the main reason for their migration, 42% were forced to shift their homes due to natural disasters.

### Impact of climate change on job sectors of RMG workers

**Fig. 3:**
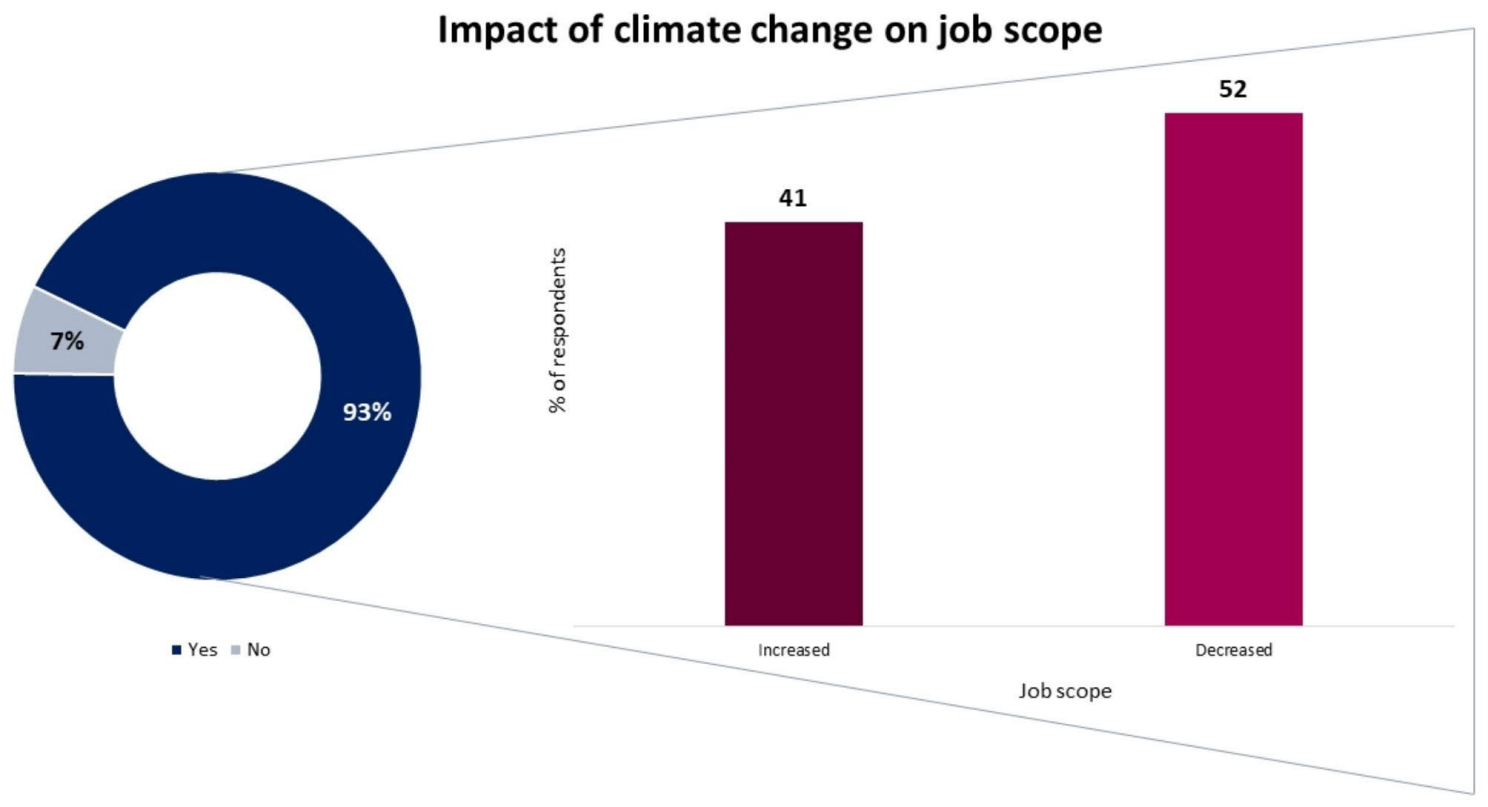
Climate change impact on job

Figure 3 depicts the impact of climate change on the job scope. About 93% agreed that the job scope had changed due to climate change, and among them, the majority mentioned that the scope had decreased (52%) from the past.

Qualitative interviews revealed that competition in the job market has increased owing to the increased flow of human resources from catastrophe-affected areas. Owners in the garment industries are getting more employees with lower salary demand.

The owners pay us less than before, as they are getting more workers at a reduced salary.

(Male RMG Worker)

### Impact of climate change on health of RMG workers

**Fig. 4:**
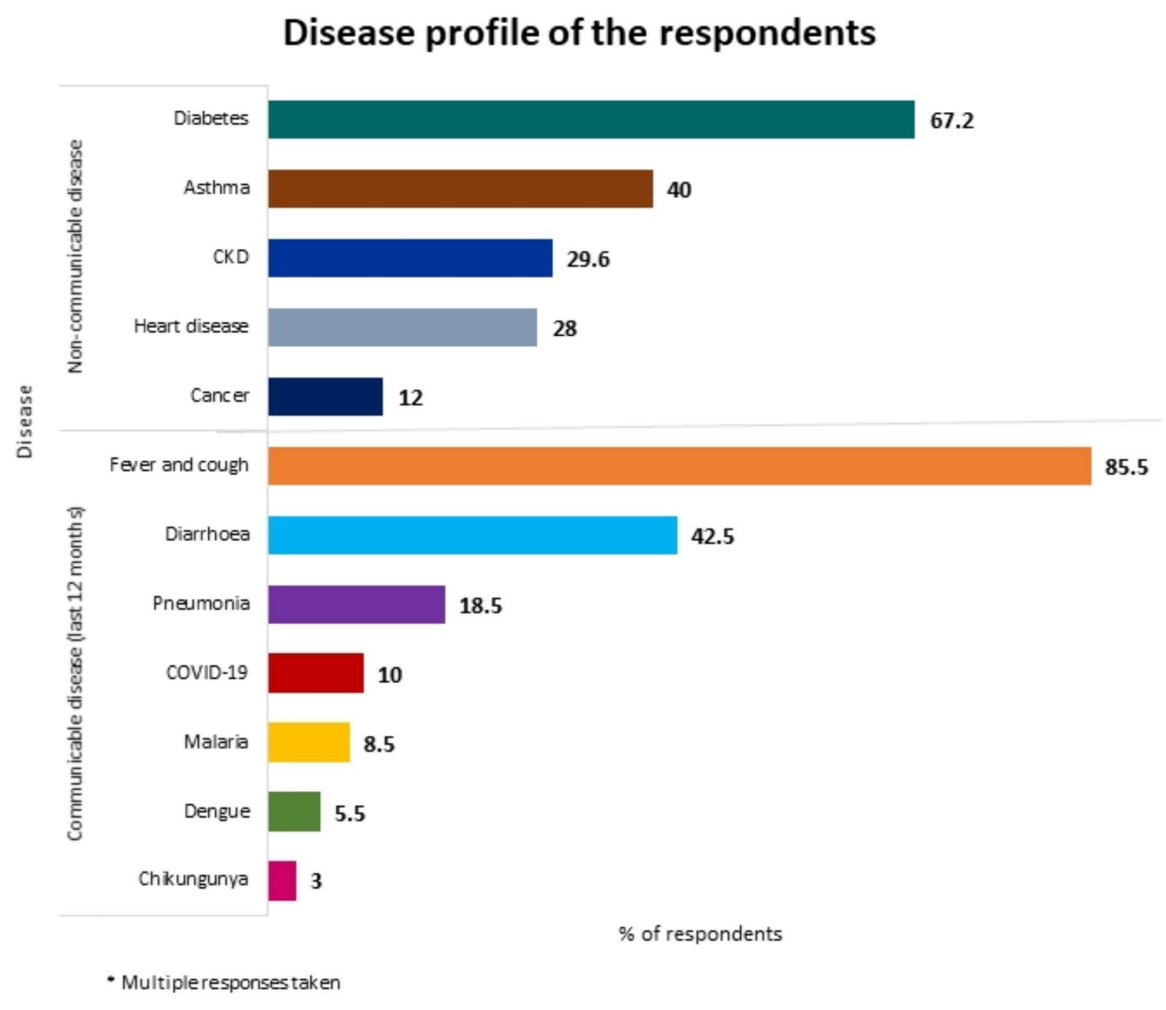
Disease profile of the RMG workers and their families

Figure 4 illustrates the disease profile of both the Readymade Garment (RMG) workers and their family members. Non-communicable diseases (NCDs), such as diabetes (around 67%), asthma (40%), chronic kidney disease (nearly 30%), heart disease (28%), and cancer (12%), were reported more frequently than communicable diseases. Over the past 12 months, most respondents and their families reported experiencing fever and cough (approximately 86%), followed by diarrhea (around 43%). COVID-19 and pneumonia jointly accounted for approximately 29% of the illnesses, while chikungunya was reported by only 3%. Less than 10% of the respondents and their families reported other diseases such as malaria (nearly 9%) and dengue (nearly 6%). Based on these findings, it is important to note that workers’ perceptions link these health conditions to the impact of climate change. This reflects their experiences and perspectives, grounded in their daily realities and observations rather than their established scientific relationships.

The respondents flagged climate change as a major contributor to such disease patterns in recent times. One male respondent mentioned,

People are becoming ill because of climate change. There are more cases of diarrhoea, fever, cough, and cold. Children and adults none are spared.

Table 5 describes the health impacts of climate change at the workplaces of RMG workers. More than half (about 59%) of the respondents mentioned that there was no extra fan/ air conditioning facility in their workplaces to address the increased heat. More than three-quarters of the respondents (78%) became sick because of increased heat while working. Approximately half of the garment factories had treatment facilities (approximately 53%) but none of these factories offered any risk allowance for sickness due to increased heat.

**Table 5:** Health impact of climate change at the workplace of the RMG workers

Variables	Frequency (n)	Proportion (%)
**Extra Fan/ Air condition facility to address increased heat**		
Present	83	41.5
Absent	117	58.5
**Became sick at the workplace due to increased heat**		
Yes	156	78
No	44	22
**Treatment facility**		
Present	83	53.2
Absent	73	46.8
**Availability of risk allowance**		
Present	0	0
Absent	200	100

Getting sick leave is a challenge for male garment workers, but the experience is quite the opposite for their female counterparts.

We become sick working in this extremely hot environment. But if we take leave they cut down our salary.

(Male RMG Worker)

We get sick leaves. We inform the supervisor If one of us gets sick during the working hour, then she gets to rest in the resting room, before getting medical help.

(Female RMG worker)

## Discussion

We conducted this mixed-method study to explore the impact of climate change on the lives and livelihoods of Readymade Garment (RMG) workers in Bangladesh. Our research has exposed the diverse challenges faced by these workers, including the adverse effects on their physical and vocational health, as well as their vulnerability to natural disasters.

It was found that climate change has had a detrimental impact on the physical and vocational health of RMG workers in Bangladesh. Communicable and non-communicable diseases have affected physical health, migration, urbanisation, job competition, and salary reduction, which have affected the vocational health of RMG workers due to climate change. The textile and clothing industries are heavily reliant on cheap labour, which is often the reason behind the existing sub-standard working conditions and underpaid and exploited workers, particularly in poor and developing countries [12]. Such conditions make them vulnerable, particularly to the negative impacts of environmental and economic change. “Climate canaries” is a used metaphor that emphasises the sufferings of ready-made garment workers because, like canaries in a coal mine, RMG workers are among the first to be affected by environmental and economic changes [33]. This metaphor indicates that the struggles and challenges faced by these workers can be viewed as early warning signs of a significantly larger hurdle [15, 34]. Workers’ choice to take individual actions to respond to and adapt to the effects of climate change may be severely constrained. Their exposure and response are controlled by the requirements of their jobs and employers; thus, they need more tailored attention and protection to respond to, adapt to, and escape the impact of climate change. In addition to the significant number of potentially affected workers, there is immense diversity in the occupational sectors that are and will continue to be disproportionately exposed to and affected by climate change [20].

Natural disasters invariably cause significant damage to infrastructure and buildings, and have a major impact on their lives and livelihoods. Many industries are located in areas that are vulnerable to natural disasters; floods, droughts, river erosion, earthquakes, storm surges, and landslides may leave workers without income or a place to live. In addition, disruptions to transportation and communication networks can impede accessibility to the industry , hampering production and commercial stability, which in turn affects workers’ livelihoods. In Bangladesh, natural disasters, such as floods and cyclones, have had a significant impact on industry. In 2017, the country was hit by severe flooding, which affected more than 41 million people and caused an estimated 2 billion USD in damage [35]. The readymade garment industry has been severely impacted, with many factories and workers’ homes being flooded and production disrupted, as flooding is one of the top six disruptors of the RMG industry in Bangladesh [36]. Studies have shown that the poor are hit the hardest by floods, as they usually live in densely populated hazard-prone locations [37, 38]. In India, similar reports have been made regarding the devastation caused by floods in the RMG industry and workers by floods [39].

The majority of the respondents did not receive any humanitarian help. Similarly, a study conducted in 2017 regarding the severe flooding of the wetland areas of Northeast Bangladesh stated that approximately one-fifth of the participants did not receive any relief or humanitarian help [40]. Inadequate relief work, hesitancy, religious and cultural mindsets, and improper distribution of relief goods were identified as possible reasons for this [41–43]. There is a research gap in addressing the effectiveness and coverage of humanitarian aid in various contexts [44].

As RMG industries are concentrated in certain regions of the country, particularly in urban areas, droughts, river erosion, and earthquakes can impact RMG factories and workers through damage or loss of infrastructure, disruption of supply chains, and displacement of workers. In addition to economic impacts, natural hazards can also affect the health and well-being of RMG workers, as they can lead to poor sanitation and hygiene conditions in factories and surrounding communities [45]. Approximately half of the participants (52.2%, 49.5%, and 45.1%, respectively) were affected by drought, river erosion, and earthquakes, and approximately 40% were affected by storm surges and landslide-inflicted damages (13%). These climate hazards can give rise to loss of land, homes, and workplace and displacement, poor living conditions, and loss of livelihoods in the population being affected [46]. It was observed that RMG workers who were affected, migrated mainly because of the unavailability of jobs. They emphasised a decrease in job scope due to climate change, similar to that in India [39]. Damage to the infrastructure, disruption of the supply chain, and shift towards automation could be possible causes for job unavailability, which could be the reasons behind the increased competition [47]. Migration related to climate change has also been observed in other countries [48, 49].

Non-communicable diseases (NCDs) had a higher overall impact on respondents than communicable diseases did. Among NCDs, diabetes affected the most respondents, followed by asthma, chronic kidney disease, heart disease, and cancer, similar to other studies included in a systematic review on the health vulnerabilities of RMG workers [45]. Although at a much lower percentage, diabetes has been reported to be among the most prevalent alongside obesity in another study conducted in Bangladesh [50]. Similar to a study conducted in the periphery of Dhaka, the most common symptoms and diseases reported were related to the respiratory system [51]. These health outcomes could be a result of continued overexposure to small particles such as cotton dust, exposure to chemicals and noise, ergonomic factors, and work stress [52]. Fever, cough, and diarrhoea were the most common symptoms amongst the respondents. A higher prevalence of diarrhoea and the common cold was observed in another study conducted on 522 RMG workers [51]. Vector-borne infections such as malaria, dengue, and chikungunya were individually reported by less than 10% of all participants in contrast to existing literature indicating that climate change and sub-standard workplace environments contribute greatly towards an increase in vector-borne diseases [46].

More than half of the survey participants reported that their workplaces lacked additional fans or air conditioning to combat rising heat, and four out of the five respondents claimed to have fallen ill due to heat while working. Several studies have articulated the excess heat and lack of proper ventilation in factories in Bangladesh [53, 54]. Half of the garment factories surveyed had medical facilities, but none provided compensation for heat-related illnesses. Similar scenarios for the existence of a medical facility but lack of compensation can be seen in the literature [51, 55]. An increase in the duration and intensity of extreme heat events can lead to increased heat-related illnesses and deaths, as shown by Smith et al. and Xiang et al. [56, 57]. People who work in indoor spaces without proper air conditioning are particularly vulnerable to extreme heat which can lead to acute health issues such as heat exhaustion, heat syncope, dehydration, and heat stroke, and give rise to as well as exacerbate chronic conditions like Chronic Obstructive Pulmonary Disease (COPD), Coronary Artery Disease (CAD), diabetes and chronic kidney disease [46, 58].

To address the potential impacts of climate change on work-related illnesses and injuries, identifying, anticipating, and controlling potential hazards that may arise as a result of climate change and the measures taken to mitigate and adapt to them are necessary. A comprehensive approach to address these challenges includes coordinated and integrated hazard recognition and response [59]. One framework that can be adapted for this purpose is the Building Resilience Against Climate Effects (BRACE) framework developed by the Centers for Disease Control and Prevention (CDC). This framework includes vulnerability assessment, preparatory action, and evaluation and can be used to reduce occupational health and safety hazards and make work environments safer [19]. Workplace strategies such as employee participation in hazard recognition and employer preparedness, prevention through design, research, and surveillance, and upholding workplace ethics and standards are among the suggested answers to a daunting problem. Further research is needed to investigate the different aspects of climate change in different groups of RMG workers in Bangladesh. Research should be conducted to discern the impacts of climate change impacts from those of other influencers.

Prior research has primarily focused on specific aspects of the impact of climate change on garment manufacturing workers, such as health and disaster vulnerability, often with a focus on particular demographic groups [12, 47, 50]. In contrast, our study adopted a comprehensive approach that examined both physical and occupational health. Although previous studies have often limited their scope to distinct localities or geographic areas, our investigation, to the best of our knowledge, represents a pioneering effort in Bangladesh [40, 41, 45, 47, 53, 54]. Our study elucidates the complex interplay among urbanisation, migration, infectious and non-communicable diseases, job competition, and wage reductions, all of which are influenced by climate change. Moreover, we transcend the immediate consequences of climate change by proposing practical alternatives including the implementation of a CDC framework. In this context, our research delves into the implications of workplace strategies aimed at enhancing occupational health and safety in the face of climate change. We emphasize the necessity for tailored interventions to address the specific needs of RMG workers facing climate change, including both communicable and non-communicable diseases, and support for those affected by natural disasters.

To mitigate the impact of climate change on RMG workers, we recommend that employers and policymakers implement measures to counter rising temperatures, ensure proper ventilation, and provide compensation for heat-related illnesses. Our proposal involves adapting the Building Resilience Against Climate Effects (BRACE) framework, originally developed by the Centers for Disease Control and Prevention (CDC), to address the effects of climate change on occupational health and safety. This perspective introduces a novel approach by leveraging established frameworks to address emerging challenges.

Although, the current study is a pioneering attempt to understand the impact of climate change in the RMG setting, it has several limitations. Self-reported data, along with low climate change knowledge in this particular scenario, may have led to reporting bias to a certain limit. The study conducted FGDs and KIIs which captured an overview; however, lacking the in-depth interview component in the data collection method created a void in the data related to personal in-depth experience on the problem at hand. Although the factories were selected randomly, individual participants were conveniently selected, creating a probability of selection bias at the sampling unit level. Keeping these limitations aside, this study calls for more focus on exploring the impact of climate change on the RMG industry, and investigating the effects of climate change through adaptive and mitigating actions. Our study focused on describing the impact of climate change on the lives and livelihoods of RMG workers and created scope for further studies focusing on the factor identification of each negative finding. These actions will allow the establishment of a doable framework to confirm that concerns about climate change are properly addressed.

## Conclusion

We found that communicable and non-communicable diseases affected the lives and migration, urbanization, job competition, and salary reduction affected the livelihood of RMG workers due to climate change in Bangladesh. At times, RMG stakeholders may separate themselves from the reality of climate change or its possible consequences in such a way that they impede adequate action. However, measures aimed at closing this gap may not be advantageous uniformly. If conceptualising the comprehensive framework is a significant and valuable attempt to reduce the impact of climate change on RMG workers, it must be properly designed. Employee participation in hazard recognition, employer preparedness, prevention through design, research, surveillance, and upholding workplace ethics and standards can be the answers to climate change problems for readymade garment workers.

## Data Availability

The datasets used and/or analyzed during the current study are available from the corresponding author on reasonable request.
